# Chemical Methods to Induce Beta-Cell Proliferation

**DOI:** 10.1155/2012/925143

**Published:** 2012-06-28

**Authors:** Amedeo Vetere, Bridget K. Wagner

**Affiliations:** Chemical Biology Program, Broad Institute of MIT and Harvard, 7 Cambridge Center, Cambridge, MA 02142, USA

## Abstract

Pancreatic beta-cell regeneration, for example, by inducing proliferation, remains an important goal in developing effective treatments for diabetes. However, beta cells have mainly been considered quiescent. This “static” view has recently been challenged by observations of relevant physiological conditions in which metabolic stress is compensated by an increase in beta-cell mass. Understanding the molecular mechanisms underlining these process could open the possibility of developing novel small molecules to increase beta-cell mass. Several cellular cell-cycle and signaling proteins provide attractive targets for high throughput screening, and recent advances in cell culture have enabled phenotypic screening for small molecule-induced beta-cell proliferation. We present here an overview of the current trends involving small-molecule approaches to induce beta-cell regeneration by proliferation.

## 1. Introduction

Both type 1 (T1D) and 2 (T2D) diabetes involve loss of beta-cell mass and function. In T1D, autoimmune destruction of beta cells results in an absolute dependence on exogenous insulin, while the peripheral insulin resistance in T2D can lead to beta-cell decompensation and failure [1]. Overall beta-cell mass is regulated by various processes, including apoptosis, differentiation, neogenesis, and proliferation. Ways to increase beta-cell mass **in** *vivo* could provide new avenues for therapeutic development.

Beta-cell replacement is therapeutic for the treatment of T1D. The Edmonton protocol for islet transplantation demonstrated that patients could achieve insulin independence one year after the procedure. However, patients required islets from at least two donors, exceeding organ supply. In a follow-up study of 36 recipients of islet treated at nine transplantation centers [2] only one-third of patients were insulin-independent after two years. These results demonstrate that increasing beta-cell mass can result in insulin independence, but that we may need methods in addition to islet transplantation to achieve this goal.

It remains unclear whether beta-cell proliferation can be exploited to treat diabetes by inducing beta-cell regeneration. Beta-cell mass is maintained at optimal levels in the body through a slow turn-over rate. In humans, it has been shown that beta-cell mass expands several fold from birth and through the first three years of childhood, but that after this initial period, beta-cell replication potential declines markedly until adulthood [3].

Work in 2004 conclusively showed that new beta cells in the mouse arise from cell division of existing beta cells and not from a stem-cell population [4]. As mentioned, study of pancreatic samples from young human donors showed that replication is indeed responsible for beta-cell expansion, but only for a short period after birth [3]. However, an analysis of donors between 7 and 66 years old found beta cells positive for the proliferation marker Ki67 in every sample tested [5]. These observations support the hypothesis that beta cells have a physiological, albeit limited, capacity to proliferate.

A critical barrier to progress in the treatment of diabetes is the lack of small-molecule drugs to induce beta-cell regeneration. Small molecule-induced beta-cell proliferation in humans could be an important way to achieve this goal; such compounds could be used to restore beta-cell mass **in** *vivo*, or alternatively provide methods for **ex** *vivo* expansion of beta-cell numbers before transplantation. We present here an overview of the current trends involving small-molecule approaches to induce beta-cell regeneration.

## 2. Physiological Mechanisms of Beta-Cell Proliferation

### 2.1. Glucose

Although beta-cell mass remains relatively constant throughout adulthood, there are a number of physiological stimuli that can promote or inhibit beta-cell proliferation; these naturally occurring conditions enable us to study them and identify novel targets for perturbation. First, glucose is a mitogen for beta-cell proliferation, with long- and short-term glucose infusion promoting beta-cell proliferation [6–8]. Human islets transplanted into nonobese diabetic mice also responded to glucose **in** *vivo* by proliferating [9]. Although the mechanism of glucose-induced proliferation has remained unclear, the role of, for example, the insulin receptor and insulin receptor substrate 2 have been shown to be important [10]. A recent study showed the importance of glycolytic flux on the stimulation of cell division [11]. After ablating the majority of beta cells in mice and inducing compensatory proliferation, the authors found that beta cells adjust their proliferation rate according to the rate of glycolysis. Glucokinase (GCK) phosphorylates glucose to glucose-6-phosphate in the first step of glycolysis. It serves as a glucose sensor and can regulate insulin secretion and beta-cell proliferation. Accordingly, mice deficient in GCK could not compensate for beta-cell ablation by proliferating, while GCK activator compounds increased this proliferation. Further evidence of GCK importance in beta-cell proliferation was provided by the identification of a rare variant (V91L) in the human glucokinase gene, in which the affinity of GCK for glucose was more than 8.5 times higher. These individuals have abnormally large islets, larger relative beta-cell area, and a high degree of proliferation, as determined by the number of Ki67-positive beta cells [12]. These studies point to GCK as an important target for small-molecule modulation.

### 2.2. Pregnancy

A well-studied phenomena associated with an increase in beta-cell mass is pregnancy. During pregnancy, the presence of the fetus creates an increased metabolic demand on the mother, resulting in increased glucose-stimulated insulin secretion, increased insulin synthesis, and a reversible increase in beta-cell replication [13, 14]. The peak of proliferation coincides with an increase in lactogen levels, such as prolactin, placental lactogen, and growth hormone [15]. Although early examination of cadaveric human islets showed an enlargement of islet size in pregnancy due to hyperplasia (from 72% beta cells per islet to 82% in pregnant women) [16], later studies showed that human islets respond to lactogens primarily by increasing insulin secretion [17] and that ductal neogenesis may in fact be occurring during pregnancy [18].

Despite the ambiguity of the adaptive response to pregnancy in humans, recent work has advanced our knowledge of this process in rodents. Work in 2007 showed that the increase in beta-cell proliferation in pregnant mice is mediated by the protein menin, encoded by the *Men1* gene [19]. Expression of menin in beta cells perfectly anticorrelates with the increase in BrdU incorporation in beta cells during pregnancy. Menin associates with the histone methyltransferase mixed-lineage leukemia (MLL), which in turn regulates expression of cell-cycle inhibitors p18 and p27. Overexpression of menin in islets causes a decompensation during pregnancy and loss of menin expression, and downstream consequences could be recapitulated in cell culture by the addition of prolactin. Consistent with these results, study of the transcription factor FoxM1 in islets found that pancreas-wide deletion of this gene resulted in loss of all proliferation during pregnancy and subsequent development of gestational diabetes [20]. These effects were accompanied by an increase in menin and p27 levels, suggesting an important role for FoxM1 and menin in the adaptation to pregnancy. Finally, another study of pregnancy in mice revealed that the expansion of beta-cell mass is at least partially regulated by serotonin signaling [21]. Tryptophan hydroxylase 1, the rate-limiting step in serotonin biosynthesis, is one of the most highly upregulated genes in pregnant islets, and the conclusion of the study was that serotonin acts as a paracrine signal to stimulate cell-cycle progression in beta cells. All these studies strongly suggest that researchers should pay particular attention to this physiological process when thinking about mechanisms to induce beta-cell regeneration.

### 2.3. Obesity

The loss of beta-cell mass has been reported in obesity and T2D [22, 23], although in some cases there is a compensatory increase in beta-cell mass [24]. Whether this adaptation is mediated by beta-cell proliferation is not well understood. Two lines of evidence point to regulation of proliferation in mouse obesity. First, the mouse model of hyperphagic obesity, *A* ^*y*^, was used to assess the levels of menin and downstream cell-cycle proteins. Relative to nonobese control littermates, *A* ^*y*^ mice have much lower mRNA and protein levels of menin, p18, and p27 [19]. This loss of menin expression correlates with increased proliferation, though it was not shown explicitly. Second, an analysis of genes regulated in nondiabetic obese *ob/ob* mice revealed that cell-cycle genes were increased in expression. In particular, FoxM1 is an important regulator of beta-cell division and is increased in these obese mice [25]. Further, overexpression of *FoxM1* stimulated [^3^H]-thymidine incorporation in whole mouse and human islets. These studies suggest that the response of beta cells to obesity may be quite similar to the response during pregnancy.

### 2.4. Aging

During aging, many tissues lose regenerative capacity, and the increase in expression of *p*16^*INK*4*a*^ has been shown to be a marker of aging [26]. This phenomenon is also observed in islets [27, 28], and aged mouse islets express far more *p*16^*INK*4*a*^ than exocrine tissue [29]. Mice with p16 deficiency had effects that depended on age: young mice were unaffected by the loss of the gene, while older mice lost any beta-cell proliferative capability. Similarly, *p*27^*KIP*1^ protein accumulates in terminally differentiated beta cells during embryogenesis [30], limiting their postnatal expansion. In humans, recent results reveal that these regulatory proteins are activated or inactivated according to the different beta-cell proliferative status. For example, p16 and p27 proteins were sporadically expressed in prenatal human beta cells, but were enriched in adult ones [31]. Thus, the effects of aging appear to be similar in rodent and human islets.

The mechanism of increased expression of cell-cycle inhibitors during aging is slowly becoming clearer. Aging in mice is associated with a decrease in expression of two repressors of the *Ink4a/Arf* locus. First, aging reduces expression of the polycomb group protein Bmi-1, resulting in increased recruitment of MLL and methylation of the locus, and increased p16 expression [32]. Accordingly, Bmi-knockout mice have increased p16 expression and decreased beta-cell mass. Second, aging decreases beta-cell expression of the histone methyltransferase enhancer of zeste homolog 2 (Ezh2). Conditional deletion of Ezh2 results in expression of p16 at a younger age and a decrease in beta-cell proliferation [33]. These studies show that the various adaptations of beta cells to the physiological stresses of hyperglycemia, obesity, pregnancy, and aging may all ultimately converge on regulation of the cell cycle in beta cells.

## 3. Cellular Targets for Proliferation

### 3.1. Cell-Cycle Proteins

From a molecular point of view, beta-cell proliferation is regulated by finely tuning protein kinases, kinase activators, or inhibitors [34–36]. Here, we will review relevant cellular proteins that could be targeted for high-throughput screening. In terms of positive regulation of beta-cell proliferation, a central role has been ascribed to cyclin D1 and D2 [37]. The role of cyclin D3 remains controversial in rodents [38], but its role seems to be more important in human pancreas [39]. Genetic manipulation through overexpression is effective in inducing beta-cell regeneration. One of the first pieces of evidence of induction in human beta cells came from overexpression of cdk-4 and cyclin D1 in islets [40]. Further, overexpression of the cyclin-dependent kinase cdk-6, either with or without cyclin D1, causes an increase in human beta-cell proliferation in both intact islets and dissociated islet cells [39, 41]. An important liability in targeting cell-cycle proteins chemically is their ubiquitous expression throughout the body. In fact, inhibition of cell-cycle kinases is an active area of research for cancer therapy [42]. It may be a daunting task to identify, for example, activators of key cell-cycle proteins that are specific only to beta cells. As discussed, many of the important physiological processes regulating beta-cell proliferation converge on the cell cycle, so this fundamental process may be too downstream for effective targeting by small molecules.

### 3.2. GSK-3*β*

Multiple lines of evidence indicate that the Wnt pathway is involved in beta-cell survival and proliferation. Activation of Wnt signaling in beta-cell lines or primary mouse islets has been shown to result in enhanced beta-cell proliferation [43–45], with upregulation of cell-cycle genes, including cyclins D1 and D2, and cdk-4 [45, 46]. Furthermore, increasing beta-catenin expression in islets causes an expansion of beta-cell mass **in** *vivo*, while upregulating axin expression, a negative regulator of Wnt signaling, blunts Wnt-stimulated gene expression and reduces beta-cell expansion [46].

**Figure 1:**
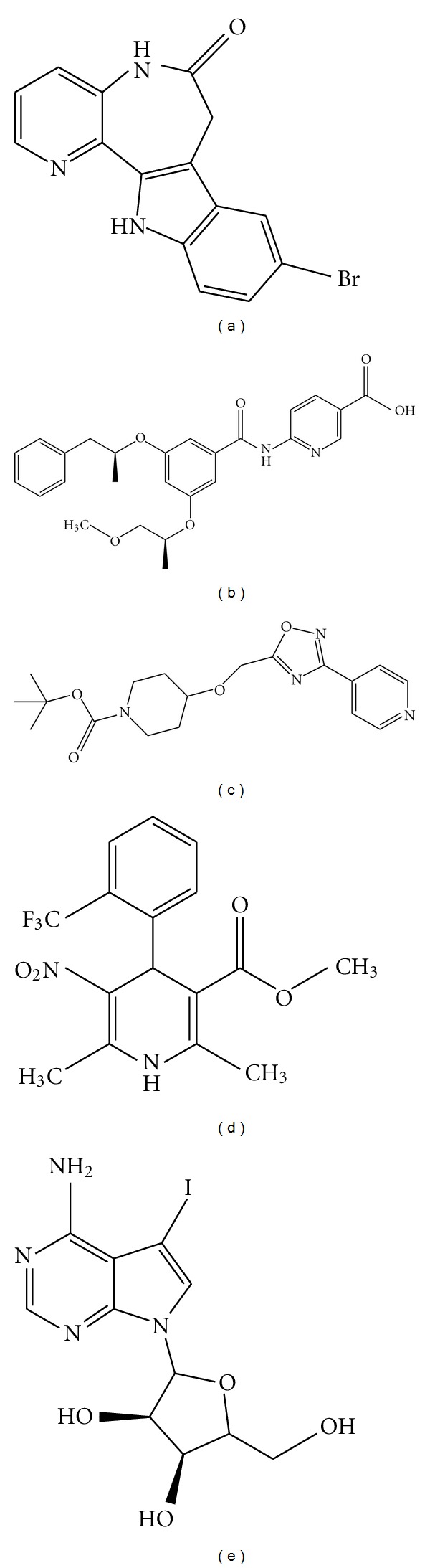
Chemical structures of small molecules reported to induce beta-cell proliferation. (a) 1-Azakenpaullone, a GSK-3*β*  inhibitor [47]. (b) GKA50, a glucokinase activator [51]. (c) PSN632408, a GPR119 agonist [52]. (d) Bay K 8644, an L-type calcium channel activator [53]. (e) 5-Iodotubercidin, an adenosine kinase inhibitor [54].

In the absence of Wnt signaling, glycogen synthase kinase-3*β* (GSK-3*β*), adenomatous polyposis coli protein, axin, and beta-catenin form components of the “destruction complex,” enabling GSK-3*β* to phosphorylate beta-catenin, targeting it for ubiquitination and degradation by the proteasome. GSK-3*β* inhibitors induce beta-cell proliferation; treatment of INS-1 cells with structurally unrelated GSK-3*β* inhibitors 1-azakenpaullone, CHIR99021, and 6-bromoindirubin-3'-oxime (BIO) increased proliferation rate in a dose-dependent manner, as measured by BrdU incorporation [47]. 1-Azakenpaullone and CHIR99021 were found to promote beta-cell replication in isolated rat islets. On the basis of these observations, novel derivatives of paullone were synthesized and assayed for their effect on beta-cell proliferation. Among those tested, 1-aza derivatives resulted in potent and selective GSK-3*β* inhibitors (Figure 1(a)). Within this series, 9-cyano-1-azapaullone (cazpaullone) showed the most efficacy in inducing beta-cell replication in both INS-1 cells and primary rat islets [48]. Work in human islets has revealed that GSK-3*β* inhibitors also promote proliferation, though the readout was total islet [^3^H]-thymidine incorporation [49]. Rapamycin also reduced this proliferation, further pointing to activation of mTOR as a potential mechanism for inducing beta-cell proliferation. With pursuit of novel GSK-3*β* inhibitors, an active area of research [50], the question of the role of GSK-3*β* in beta cells will be answerable with ever more selective and potent compounds.

### 3.3. Glucokinase

As mentioned above, glucokinase (GCK) phosphorylates glucose to glucose-6-phosphate in the first step of glycolysis. It is the predominant hexose kinase in beta cells and plays an important role as a glucose sensor in beta cells. When fed a high-fat diet, wild-type mice showed marked beta-cell hyperplasia, while GCK heterozygous mice could not sufficiently increase beta-cell replication [55], leading to the conclusion that glucokinase plays a critical role in inducing beta-cell replication. To address this aspect, GCK^+/−^ mice were fed with high-fat diet in the presence or absence of a glucokinase activator (GKA) for 20 weeks [56]. No significant differences in beta-cell proliferation were seen after chronic treatment, but after an acute 3-day treatment with GKA, beta-cell proliferation was markedly stimulated.

Potent proliferative effects of the glucokinase agonists GKA50 (Figure 1(b)) and LY2121260 were also observed in the INS-1 cell model [51]. Interestingly, GKA50, but not LY2121260, also prevented apoptosis in INS-1 cells under chronic high-glucose concentrations, probably by increasing the levels of glucokinase protein itself. Glucokinase agonists could play important role in promoting beta-cell proliferation and preventing apoptosis, and novel compounds are actively being sought for these purposes [57].

### 3.4. GLP-1

Although much study has focused on glucagon-like peptide 1 (GLP-1) and its role in diabetes, important questions remain unanswered. For example, the ability of exendin-4 to induce beta-cell proliferation in rodents is well established [58–61], but whether this effect also occurs in humans has not yet been demonstrated [62]. A recent report showed an increase in the replication of human beta cells transplanted into mice treated with exendin-4, but only when the islets were from young donors [63]. Such observations raise questions about the differences, in terms of the molecular machinery and islet physiology, existing between murine models and humans. On the other hand, the fact that GLP-1 appears to activate Wnt in a TCF7L2-dependent manner [64] is encouraging for a translation to human biology, as single-nucleotide polymorphisms in TCF7L2 provide the strongest genetic associations with T2D [65]. It may be the case that such effects of GLP-1 activation are only observable when administered to a whole organism, and not in cell culture alone.

There is a great scientific and commercial interest in identifying GLP-1 mimetics and inhibitors of dipeptidyl peptidase 4, which degrades GLP-1 **in** *vivo* [66]. These compounds promote activation of the GLP-1 G protein-coupled receptor to stimulate insulin secretion and inhibit glucagon secretion, and also have the potential to increase beta-cell mass (see [67] for an extensive review). Despite some interesting successes, the development of small-molecule agonists of the GLP-1 receptor is still quite challenging and not yet fully developed.

### 3.5. GPR119

G protein-coupled receptor 119 (GPR119) is another attractive target for T2D therapy [68]. GPR119 was first identified as an orphan GPCR in various mammalian species [69]. This receptor is predominantly expressed in beta cells [70] and GLP-1-secreting intestinal L-cells [70]. The phospholipids lysophosphatidylcholine and oleoylethanolamide (OEA) are natural endogenous ligands for GPR119 [71, 72]; each of them increases intracellular cAMP levels and results in glucose-dependent insulin secretion. Gao et al. [52, 73] evaluated the effects of OEA and synthetic agonists (PSN632408 and AR231453) on murine beta-cell replication (Figure 1(c)) and found that all were able to increase the number of replicated beta cells compared to control animals. Similar results have been reported for other GPR119 agonists: AS1535907 [74] and APD597 [75]. These results provide interesting preliminary evidence that activating GPR119 may be a feasible strategy for treating T2D.

## 4. Chemical Screening and Beta-Cell Proliferation

In the past few years, attempts have been made to develop assays for high-throughput screening (HTS) to identify small-molecule inducers of pancreatic beta-cell expansion. One of the first studies reported screening a heterocyclic library of 850,000 compounds for proliferation of the reversibly immortalized mouse cell line R7T1 [53]. This beta-cell line was immortalized using the SV40 T antigen under the control of the Tet-On system, such that the cells proliferate in presence of tetracycline but undergo growth arrest upon its withdrawal [76]. These cells express characteristic beta-cell markers (insulin 1, insulin 2, and Pdx1), secrete insulin, and restore euglycemia in streptozotocin-treated mice. Although not a true mimic of **in** *vivo* quiescent beta cells, this system can be considered a reasonable proxy to identify reentry into the cell cycle of growth-arrested cells as measured by intracellular ATP levels. To rule out false-positive tetracycline mimetics, the Tet-Off immortalized beta-cell line *β*TC-Tet, which proliferates only in the absence of Tet [76], was used as a counter screen.

Several structurally diverse, active compound classes were identified, including phorbol esters, dihydropyridines (DHP), and thiophene pyrimidines. In particular, the thiophene pyrimidines appeared to stimulate beta-cell proliferation by activating the Wnt signaling pathway. A piperazinyl derivative showed a dose-dependent induction of R7T1 proliferation with an IC_50_ of about 1.1 *μ*M. The same compound was active in MIN6 and HIT-T15 beta-cell lines and in primary rat beta cells. Interestingly, this molecule turned out to be a potent GSK-3*β* inhibitor.

The dihydropyridine (DHP) class identified in this screen acts on L-type calcium channels. In beta cells, calcium channels play a key role in controlling glucose-stimulated insulin secretion and insulin production [77], and polymorphisms in some calcium channel-encoding genes are associated with both T1D and T2D [78–80]. However, it is also possible that these mutations lead to proliferative defects in beta cells. In fact, the LTCC_a1D_ subunit knockout displayed a significant reduction of postnatal beta-cell proliferation [81], suggesting that calcium channel signaling is necessary for beta-cell replication. The most potent derivative identified (Bay K 8644; Figure 1(d)) resulted in a significant dose-dependent increase in proliferation of growth-arrested R7T1, HIT-T15 and MIN6 beta-cell lines, and primary rat beta cells. Data showing induction of human beta-cell proliferation, however, were less conclusive, suggesting that further study is required to identify inducers that are active in human islets.

Recently, we have developed a human islet cell-culture system [82] to screen for inducers of beta-cell proliferation directly in primary human cells. While very challenging, we have used this system to measure the percentage of insulin^+^/Ki67^+^ cells after compound treatment in 384-well plates. The major limitation is acquiring an adequate supply of human islets. A proof-of-concept screen using primary rat islets has also been reported [54]. Dissociated islets were seeded in 96-well plates and treated with a library of ~850 cell-permeable bioactive compounds. The primary screen measured the percentage of PDX1^+^/Ki67^+^ cells. Two adenosine kinase inhibitors: 5-iodotubercidin (5-IT; Figure 1(e)) and ABT-702 were screening hits, and both induced a two- to three-fold increase of the percentage of the PDX1^+^/Ki67^+^ cells compared to DMSO-treated controls. **In** *vivo* studies were carried out using ABT-702 due to its longer half-life; intraperitoneal injection of 21 mg/kg ABT-702 resulted in a two-fold increase in BrdU incorporation in PDX1^+^ cells, but had no effect on either exocrine cells or hepatocytes. No data were presented in human islets, so it remains to be seen whether adenosine kinase plays an important role in human beta-cell proliferation.

Adenosine kinase is highly expressed in the liver and pancreas [83] and plays an important role in cellular metabolism [84]. To understand why adenosine kinase inhibitors induce beta-cell replication, islet cultures were treated with both 5-IT and various replication-pathway inhibitors. Only the p38 mitogen-activated protein kinase (MAPK) inhibitor SB203580 increased 5-IT-dependent beta-cell replication, while rapamycin and the PI3K inhibitor wortmannin each suppressed beta-cell replication, suggesting 5-IT promotes beta-cell replication in an mTOR-dependent manner. The authors also observed an interesting link with glucose and GLP-1; the combination of 5-IT with high glucose, GLP-1, or exendin-4 induced the replication rate four- to five-fold. These results point to adenosine kinase as yet another possible target for beta-cell regeneration.

## 5. Future Directions: Cellular Targets for Screening

An impression that comes from the many studies performed on beta-cell proliferation is that there seem to be as many possible targets as there are studies. Because a potential goal in diabetes regenerative medicine is to induce proliferation of beta cells, it is important to find mechanisms that are very specific for beta cells. Otherwise, the risk for any compound that is identified is that it globally induces cell division, a potentially catastrophic consequence. In order to identify selective small molecules, it is likely that future directions should involve processes that are relatively specific to beta cells.

The adaptive response of beta cells to pregnancy provides several possibilities for cellular targets. First, serotonin is highly upregulated in beta cells during pregnancy [21]. Thus, activation of serotonin biosynthesis or signaling may be a novel and specific way to induce beta-cell proliferation. Increased knowledge of the mechanisms by which serotonin increases proliferation will help in this quest. Second, the protein menin is highly downregulated during pregnancy [19]. Menin is interesting because of its specificity towards beta cells, and its ability to act as a switch that can turn on or off beta-cell proliferation. Recently, these unique properties have been assessed in a hyperglycemic diabetic mouse model, where acute and temporally controlled deletion of *Men1* improves preexisting hyperglycemia in streptozotocin-treated mice, and reverses glucose intolerance in high-fat diet-fed mice by increasing proliferation of beta cells [85]. A very recent study reports screening for disruption of the menin-MLL complex [86], so it will be of great interest to determine whether these compounds also increase beta-cell proliferation.

The prospect of targeting menin and/or MLL highlights the likely important role epigenetics will play in understanding beta-cell proliferation in the future. There is accumulating evidence that beta cells regulate their growth and fate by epigenetic mechanisms [32, 87]. Thus far, modulation of epigenetic status has not been fully exploited in beta-cell physiology and could represent an array of novel therapeutic targets. Indeed, some interesting examples of the potential of this approach are already available, particularly histone deacetylase inhibitors [88, 89]. A number of other cellular targets provide attractive models, including mTOR activation, as discussed above, and Pax4 activation [90].

Most of the studies in beta-cell biology, including the more recent high-throughput screening examples, rely heavily on murine animal and cell models. Although these studies provide the foundation for many advances in beta-cell research, unfortunately they often do not translate to similar results when applied to humans. The greatest challenge facing beta-cell regeneration approaches, particularly proliferation, is the requirement for treatments and mechanisms to work in both rodents and humans. As we understand more about the physiological proliferative behavior of human beta cells, we can start to identify the molecular switches that could be potentially used to foster the proliferation of beta cells in humans.
